# Dual *Lactiplantibacillus plantarum*-Derived Postbiotics Reduce Pathogens and Preserve the Quality of Goldenberry (*Physalis peruviana* L.) During Storage

**DOI:** 10.3390/foods15101830

**Published:** 2026-05-21

**Authors:** Diana Molina, Pamela Reyes, Yuleissy Cuamacas, Evelyn Angamarca, Clara Ortega, Renato Centeno, Gabriela N. Tenea

**Affiliations:** Biofood and Nutraceutics Research and Development Group, Faculty of Engineering in Agricultural and Environmental Sciences, Universidad Tecnica del Norte, Ibarra 100105, Ecuador; dmolinap@utn.edu.ec (D.M.); pcreyes@utn.edu.ec (P.R.); yncuamacasc@utn.edu.ec (Y.C.); elangamarca@utn.edu.ec (E.A.); cgortegab@utn.edu.ec (C.O.); rxcentenoa@utn.edu.ec (R.C.)

**Keywords:** postbiotics, antimicrobials, *Physalis peruviana*, *Lactiplantibacillus plantarum*, fruit quality

## Abstract

Microbial contamination of fresh fruits remains a major food safety concern due to the ability of pathogenic bacteria to persist on fruit surfaces during storage. This study evaluated the antimicrobial efficacy of ExAF-E1, a postbiotic formulation derived from *Lactiplantibacillus plantarum* strains UTNGt28L and UTNGt2, against multidrug-resistant *Escherichia coli* L1PEag1 and *Staphylococcus epidermidis* L4MStp5 on goldenberry (*Physalis peruviana* L.). Fruits were artificially contaminated, treated, and stored for 7 days at room temperature (RT) and refrigerated (4 °C), with analyses conducted in quadruplicate. At RT, ExAF-E1 significantly reduced total aerobic counts (TAC) and pathogen loads (*p* < 0.05), achieving early reductions of ~0.4–0.5 log CFU/g in TAC and ~1.0–1.5 log CFU/g in pathogens, with inhibition maintained through day 7. In contrast, the commercial disinfectant (CD) showed transient reductions, with microbial levels not significantly different from the control at later stages (*p* > 0.05). Under refrigeration, ExAF-E1 produced greater and persistent reductions, reaching ~1.0–1.2 log CFU/g in TAC and ~1.5–2.5 log CFU/g in pathogens by day 7 (*p* < 0.05), whereas CD exhibited strong initial reductions followed by partial regrowth. Fruit quality parameters (pH, TA, TSS, TPC, AOX, AAC) were not significantly affected by treatments (*p* > 0.05). Ultrastructural analyses using transmission and scanning electron microscopy revealed disruption of bacterial cell envelope integrity, including membrane damage, cytoplasmic leakage, and morphological deformation. These findings demonstrate that ExAF-E1 provides rapid and sustained antimicrobial activity under both storage conditions while preserving fruit quality, supporting its application as a postharvest strategy for improving the microbial safety of fresh produce.

## 1. Introduction

Postharvest fruit losses represent a major global challenge, largely driven by microbial spoilage that accelerates senescence, compromises organoleptic and nutritional quality, and increases food safety risks [1]. Conventional preservation strategies rely heavily on synthetic fungicides and chemical sanitizers; however, their continued use raises toxicological and environmental concerns, contributes to the emergence of resistant microbial populations, and is increasingly constrained by regulatory policies [2]. These limitations have intensified the search for safe, sustainable, and biologically derived alternatives for postharvest disease control.

In this context, microbial-based preservation strategies have gained increasing attention, particularly those based on beneficial microorganisms and their derivatives. Among these, lactic acid bacteria (LAB) are well known for their ability to produce antimicrobial compounds during growth, contributing significantly to food safety and shelf-life extension. In addition, certain LAB species are recognized as probiotics, defined as live microorganisms that confer a health benefit to the host when administered in adequate amounts [3].

Beyond viable cells, growing interest has shifted toward non-viable microbial preparations, commonly referred to as inactivated probiotics or paraprobiotics, which retain biological functionality through structural components such as cell wall fragments and intracellular constituents [4,5]. In parallel, the metabolites produced by probiotic microorganisms are increasingly exploited in food applications [5]. These metabolites, referred to as postbiotics, offer distinct advantages over live microbial formulations, particularly in terms of enhanced stability, safety, and regulatory acceptance [5,6].

Postbiotics are bioactive preparations derived from microorganisms that include both extracellular metabolites (exo-metabolites) accumulated in cell-free supernatants (CFS) after removal of microbial cells and components of inactivated bacteria, such as cell wall fragments, bacterial lysates, and exopolysaccharides [7]. These compounds include organic acids (e.g., lactic and acetic acid), hydrogen peroxide, diacetyl, and antimicrobial peptides such as bacteriocins, which are effective against a broad range of spoilage microorganisms and foodborne pathogens [8]. Their antimicrobial activity involves multiple mechanisms, including cytoplasmic acidification, membrane disruption, induction of oxidative stress, and interference with microbial signaling pathways [9]. The direct application of these extracellular fractions as antimicrobial formulations has gained increasing interest, as they provide bioactive compounds facilitating their use in food preservation [10]. In contrast to conventional chemical sanitizers, these compounds act through mechanisms such as membrane disruption, enzymatic activity, and metabolic interference, potentially enabling selective suppression of undesirable microorganisms while preserving beneficial microbiota [11,12]. Despite increasing evidence supporting their antimicrobial efficacy, the impact of CFS-based interventions on fresh product-associated microbial communities remains insufficiently understood [13]. This limitation is especially relevant for highly perishable fruits, where microbial interactions directly influence shelf life, safety, and quality attributes.

Goldenberry (*Physalis peruviana* L.), also known as uvilla, uchuva, or cape gooseberry, is an Andean fruit cultivated at elevations between 1000 and 3000 m above sea level in countries such as Ecuador, Peru, Colombia, and Bolivia [14]. Increasing international demand has driven the expansion of Ecuadorian exports to markets in North America and Europe [15]. However, goldenberry is highly perishable due to its high moisture content and nutrient-rich composition, which favor microbial growth during storage. Postharvest losses of approximately 21% have been associated with inadequate storage conditions and fungal contamination, including *Cladosporium*, *Phomopsis*, *Pestalotia*, *Botrytis cinerea*, and *Alternaria* species [16,17,18]. More broadly, microbial spoilage contributes to postharvest losses of up to 33% in fresh fruits and vegetables [19,20], with significant economic and logistical implications. Beyond its economic importance, goldenberry is valued for its nutritional composition, including vitamins A, C, and E, carotenoids, and flavonoids [21,22]. However, its susceptibility to microbial contamination also raises food safety concerns. Previous work identified a multidrug-resistant *Escherichia coli* strain (L1PEag1) on the surface of ready-to-eat goldenberries, indicating that fresh fruits can act as reservoirs of antibiotic-resistant bacteria [23]. These observations underscore the need for microbial control strategies that reduce contamination while preserving fruit quality, particularly in regions with limited postharvest infrastructure [24,25,26].

Here, we investigate the antimicrobial effect of LAB-derived postbiotics isolated from fruit-associated LAB strains, including *Lactiplantibacillus plantarum* UTNGt2 (Gt2), from white cocoa (*Theobroma grandiflorum*), and *L. plantarum* UTNGt28L (Gt28L), from *Chrysophyllum caimito* [27,28]. Their distinct ecological origins enable assessment of strain-dependent metabolic variability and its contribution to antimicrobial activity. Among previously developed LAB-derived formulations [27], ExAF-E1-comprising CFS from Gt28L and Gt2 (3:1, *v*/*v*) exhibited strong in vitro inhibition of multidrug-resistant *E. coli* L1PEag1 and *Staphylococcus epidermidis* L4MStp5. However, its efficacy on fruit surfaces under postharvest conditions remains poorly characterized. Accordingly, this study evaluates the antimicrobial performance of ExAF-E1 on artificially contaminated fruits stored under different conditions, using a commercial disinfectant (CD) as a comparator. Fruit quality was assessed via physicochemical parameters (pH, titratable acidity [TA], total soluble solids [TSS]) and functional attributes (total phenolic content [TPC], antioxidant capacity [AOX], and anthocyanin content [AAC]). Structural effects on bacterial cells were examined via transmission and scanning electron microscopy (TEM, SEM). This work provides an integrated assessment of microbial inactivation, quality preservation, and ultrastructural alterations associated with LAB-derived postbiotic applications on fresh produce.

## 2. Materials and Methods

### 2.1. Bacterial Strains

*L. plantarum* UTNGt28L (Gt28L) (BioProject: PRJNA1116628, BioSample: SAMN49560224) and *L. plantarum* UTNGt2 (Gt2) (BioProject: PRJNA705232, BioSample: SAMN18053630), previously isolated and characterized were routinely grown in MRS broth (# 1.10661, Merk Millipore, Burlington, MA, USA). The MDR *E. coli* L1PEag1 strain (GenBank Genome Assembly GCF_036870985.1) and *S. epidermis* L4MStp5 (laboratory use, isolated from ready-to-eat goldenberries), were grown in Luria Bertani (LB) (# L3397, Sigma Aldrich Inc., Saint Louise, MO, USA) and Brain Heart Infusion (BHI, # 53286, Merk Millipore, Burlington, MA, USA) broth culture media. All microorganisms were stored at −80 °C in 20% glycerol (*v*/*v*).

### 2.2. ExAF-E1 and Pathogen Cocktail Preparation

The ExAF-E1 formulation, consisting of a 3:1 (*v*/*v*) mixture of CFS from Gt28L and Gt2, was prepared as previously described [27]. Briefly, LAB strains were cultured in MRS broth at 37 °C for 27 h under aerobic conditions. Cultures were centrifuged at 13,000× *g* for 30 min at 4 °C to remove bacterial cells, and the resulting supernatants were sequentially filtered through 0.22 µm PTFE syringe filters (# STF020025H, Chem Lab Inc. Moreno Valey, CA, USA) to ensure sterility. The sterile CFS were stored at 4 °C until use. Prior to application, the combined ExAF-E1 preparation was adjusted to pH 6.0 using sterile NaOH, followed by lyophilization to obtain a stable dry fraction. For preparation of the pathogen cocktail, 1 mL of overnight culture of each strain was inoculated into 100 mL of LB or BHI broth and incubated at 37 °C for 24 h. Cells were harvested via centrifugation at 6000 rpm for 5 min, washed once with sterile distilled water, and standardized to an optical density of OD_605_ = 1.0. Bacterial pellets were resuspended in sterile water to a final concentration of 1 × 10^8^ CFU/mL, and equal volumes (1:1, *v*/*v*) of each suspension were combined to prepare the inoculum cocktail used for artificial contamination of goldenberries.

### 2.3. Pathogen Colonization Dynamics on Goldenberry Surface

Ready-to-eat fruits without calyx were obtained from a local farm and selected at ripeness stage four according to the Ecuadorian standard INEN 2485:2009 [29], which defines maturity based on a combination of physical attributes. Selection criteria included uniform peel coloration (yellow to orange), absence of visible defects or damage, and consistency in fruit size and firmness. To minimize variability, all fruits were obtained from the same supplier and were manually sorted prior to experimentation to ensure homogeneity across samples. In addition, physicochemical parameters (pH, TSS, and TA) were monitored during storage, providing further validation of the selected maturity stage and consistency among samples. Prior to experimentation, fruits were disinfected through immersion in 5% sodium hypochlorite for 5 min, followed by two rinses with tap water and two with distilled water (30 s/washing). Samples were air-dried at room temperature for approximately 1 h under a biosafety cabinet to eliminate residual moisture. A subset of unwashed fruit samples (UWS) was weighed and stored in aerated trays to establish baseline microbiological counts at days 0 and 7. Colonization dynamics were assessed using an artificial inoculation model following a method described previously with some modifications [30]. The experiment was conducted using four independent replicates, each corresponding to a separate fruit recollection (biological replicate). Within each replicate, all treatments (exposure times and storage conditions) were included to account for variability associated with fruit origin and handling.

The experimental design consisted of 20 fruits per treatment condition across three exposure times and four independent biological replicates (240 fruits for inoculation trials), with additional 80 fruits allocated to control groups. Fruits were placed in sterile zip-lock bags and inoculated with a pathogen cocktail standardized to OD_605_ = 1.0 [31]. This inoculum level (approximately 10^7^–10^8^ CFU/mL) was selected to ensure a consistent and reproducible microbial load, commonly applied in artificial contamination studies, and to simulate a worst-case yet realistic postharvest contamination scenario resulting from cross-contamination events. Agitation was performed at 80 rpm for 10, 45, or 75 min to evaluate time-dependent bacterial attachment to the fruit epidermis. These intervals were selected to determine the minimum contact time required for stable skin colonization. Following exposure, fruits were dried for 1.5 h in a pathogen-designated laminar flow cabinet and processed for surface microbial enumeration to identify the earliest time point of consistent attachment. The drying step was standardized across all treatments and did not constitute a storage period or allow significant bacterial growth prior to further processing. Inoculated controls were stored at 4 °C and at room temperature, and bacterial loads (CFU/g) were quantified on days 1, 2, 3, 5, and 7 post-inoculation.

### 2.4. Treatment of Artificially Contaminated Goldenberries with ExAF-E1

**Figure 1:**
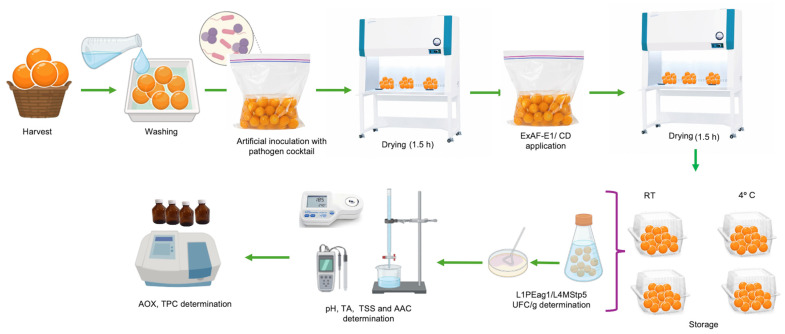
Workflow for the preparation, inoculation, treatment, microbiological and physicochemical analysis of goldenberries. Legend: ExAF-E1: LAB-derived postbiotic formulation; CD: commercial disinfectant; TA: titratable acidity, AAC: acid ascorbic content; AOX: antioxidant capacity; TPC: total polyphenols content; TSS: total soluble solids.

A schematic overview of the experimental design is shown in Figure 1.

Following determination of the optimal attachment time (75 min; Section 2.3), artificially contaminated fruits were subjected to post-inoculation treatments. A total of 600 fruits (20 fruits × three replicates × two treatments × five sampling intervals) were included in this assay. Contaminated fruits were assigned to two treatment groups: ExAF-E1 and a commercial disinfectant (CD; Star Bac Domestic, Uberlândia, MG, Brazil), a bactericidal solution for fruits and vegetables containing citric acid, benzalkonium chloride, glycerol, propylene glycol, glucose, fructose, and water. Lyophilized ExAF-E1 (20 mL) was reconstituted in sterile distilled water to a final volume of 200 mL, and fruits were immersed in the solution. The CD washing solution was prepared according to the manufacturer’s instructions, and fruits were treated using the same immersion procedure. In addition, a sterile distilled water immersion (washed samples, WS) was included as a control to account for the mechanical removal of microorganisms during washing. After a 10 min exposure period in the biosafety cabinet, fruits were air-dried for 1.5 h under aseptic laminar flow conditions with low airflow to ensure complete removal of surface moisture. Fruits were then transferred to sterile plastic trays. Samples were stored either at room temperature (RT) or 4 °C and monitored over a 7-day period. For comparative purposes, inoculated but untreated fruits served as positive controls (C), while WS and unwashed samples (UWS) with no pathogen inoculated were included as negative controls to assess background microbiota and handling effects.

### 2.5. Bacteriological Analysis

Microbiological analyses were conducted on days 0, 1, 3, 5, and 7 post-treatments. For each condition, 20 goldenberries were individually placed in sterile bags containing 1% peptone water and incubated at 37 °C for 2 h. Bacterial cells were recovered via centrifugation at 8000× *g* for 5 min and resuspended in 10 mL of 1× PBS. Microbial quantification was performed using both selective and non-selective culture media. Total aerobic counts (TAC), including both inoculated pathogens and native microbiota, were determined using Plate Count Agar (PCA, Sigma Aldrich Inc., Saint Louise, MO, USA). For specific enumeration, aliquots (100 μL) were plated on Chromocult Coliform agar (Merck Millipore, Kenilworth, NJ, USA) to quantify *E. coli* L1PEag1, while Brilliance Staph 24 Agar (Oxoid Limited, Hampshire, UK) was used for the detection and enumeration of *Staphylococcus* spp. following ISO 6888-1:1999 guidelines [32]. All experiments were performed in triplicate, and results were expressed as CFU/g.

### 2.6. Fruit Quality Evaluation

Fruit quality was assessed on treated and untreated samples over storage periods. For each treatment and sampling interval, fruits were pooled and homogenized using a laboratory blender to obtain a representative and uniform juice/extract suitable for physicochemical and functional assays. pH was determined using an electrode immersion pH meter (S210, Mettler Toledo, Columbus, OH, USA). TTA was measured by titrating 25 mL of fruit juice with 0.1 N NaOH using phenolphthalein as an indicator [33], while AAC was expressed as a percentage per 100 mL of juice. TSS was recorded using a digital refractometer [33]. TPC was quantified via the Folin–Ciocalteu method with gallic acid as the standard, expressed as mg gallic acid equivalents per gram of sample (mg GAE/g), and calibrated with gallic acid standards (0–200 μg/mL). AOX was evaluated through DPPH radical scavenging activity, with Trolox as the reference standard [34]. AAC was determined using the 2,6-dichloroindophenol titrimetric method [33]. All analyses were performed in triplicate on freshly prepared fruit extracts.

### 2.7. Effect of ExAF-E1 on Bacterial Integrity and Ultrastructural Changes

The effect of ExAF-E1 on L1PEag1 and LM4Stp5 was examined using both TEM and SEM. Exponentially growing cells (1 × 10^6^ CFU/mL) were treated with 1× MIC concentrations of ExAF-E1 for 6 h at 37 °C. For TEM, samples were fixed, embedded, ultrathin-sectioned, and stained with uranyl acetate and lead citrate before imaging on a Tecnai G2 F20 transmission electron microscope (FEI, Hillsboro, OR, USA); ten random sections per treatment were analyzed. For SEM, treated and untreated cells were washed, air-dried, fixed in 2.5% glutaraldehyde at 4 °C, dehydrated through graded ethanol, critically point–dried, mounted, and sputter-coated with ~24.5 nm gold using a DENTON VACUUM Desk IV coater. (Moorestown, NJ, USA). Surface morphology was examined using a JSM-6490 LV scanning electron microscope (JEOL, Peabody, MA, USA) under high vacuum with secondary electron detection.

### 2.8. Statistical Analysis

The mean ± standard deviation was used to present the results. Tukey’s post hoc test and the Kruskal-Wallis one-way analysis of variance (non-parametric) were employed to identify significant differences between the means (SPSS version 10.0.6, US). The cutoff value for statistical significance was set at *p* < 0.05. Furthermore, a PCA of microbial, physicochemical variables (pH, TSS, TTA) and AAC was performed on both treated and untreated fruits during storage. Graphs were prepared using GraphPad Prism v. 10.0 (GraphPad Software, San Diego, CA, USA).

## 3. Results and Discussions

### 3.1. Microbial Colonization Dynamics as a Function of Contact Time and Storage Temperature

**Figure 2:**
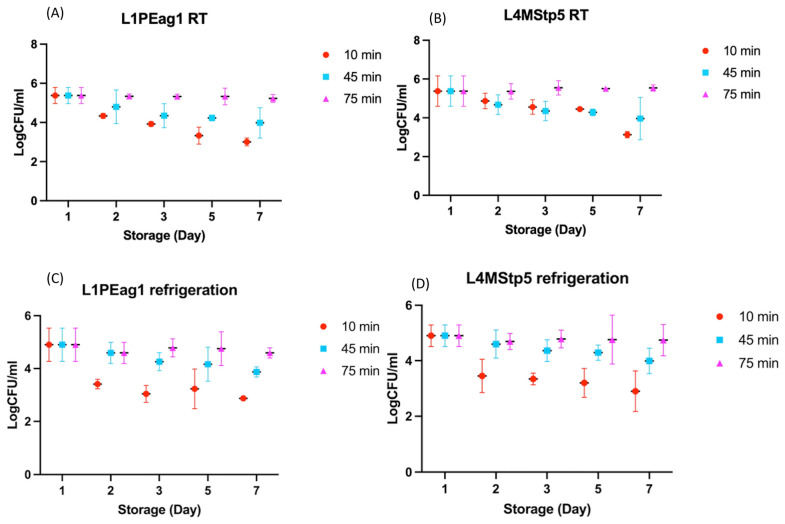
Effect of pathogen–fruit contact time (10, 45, and 75 min) and storage temperature on the persistence of L1PEag1 (*E. coli*) and L4MStp5 (*S. epidermidis*) on goldenberry surfaces during 7 days of storage. Panels: (**A**) L1PEag1 at RT, (**B**) L4MStp5 at RT, (**C**) L1PEag1 at 4 °C, and (**D**) L4MStp5 at 4 °C. Data are expressed as log CFU/g (mean ± SD, n = 4).

Microbial persistence on goldenberry skin was strongly influenced by pathogen–fruit contact time prior to storage (Figure 2).

Short exposure (10 min) resulted in substantial population declines at both storage temperatures. Under RT, L1PEag1 decreased from 5.38 to 3.01 log CFU/g and L4MStp5 from 5.38 to 3.13 log CFU/g after 7 days (Figure 2A,B), corresponding to reductions of 2.37 log (~99.6%) and 2.25 log (~99.4%), respectively. A comparable pattern was observed at 4 °C (Figure 2C,D), where populations declined from 4.90 to 2.87 log CFU/g for L1PEag1 and to 2.90 log CFU/g for L4MStp5, representing reductions of 2.03 log (~99.1%) and 2.00 log (~99.0%). These results indicate that brief contact leads to weakly established populations that progressively decline during storage. Increasing the exposure time to 45 min resulted in smaller reductions, indicating partial stabilization of skin-associated cells. After 7 days, populations at RT remained at 3.98 and 3.96 log CFU/g for L1PEag1 and L4MStp5 (1.40–1.42 log reduction), while under refrigeration, they stabilized at approximately 3.87–3.99 log CFU/g (0.9–1.0 log reduction). In contrast, prolonged contact (75 min) resulted in high persistence. At RT, both pathogens remained highly stable, maintaining populations above 5.2–5.5 log CFU/g throughout storage with negligible variation (≤0.1 log). Under refrigeration (4 °C), only slight declines were observed, with populations ranging from 4.59 to 4.74 log CFU/g by day 7, corresponding to modest reductions of 0.16–0.31 log.

The observed patterns may be consistent with the transition from reversible adhesion to more stable attachment during the early stages of bacterial colonization on fruit surfaces [35]. At short exposure times, bacterial cells are likely only loosely associated with the epidermis, mainly through weak physicochemical interactions, which may make them more vulnerable to environmental stress and antimicrobial treatments [36,37]. With increasing contact time, these interactions may become more stable, potentially allowing cells to initiate microcolony formation and produce extracellular polymeric substances (EPS). The accumulation of EPS has been associated with stronger surface attachment, reduced accessibility of antimicrobial compounds, and the development of structured biofilm communities that enhance persistence on the fruit surface [38]. This progression may help explain the differences in treatment efficacy observed across exposure times. The higher reductions obtained after short exposure (Figure 2A–D) suggest that early-stage cells can be more effectively removed or inactivated before EPS-mediated protection is established. In contrast, the lower reductions observed after 45–75 min may indicate that once cells begin to form microcolonies and produce EPS, their tolerance increases, possibly due to limited penetration of antimicrobial compounds and localized protective effects within the matrix [39,40].

Refrigeration slowed microbial activity but did not alter the influence of the initial colonization phase, suggesting that early attachment may play a key role in subsequent persistence [36]. Since bacterial attachment to fresh produce can occur within 30–60 min [35], the time between contamination and treatment becomes critical. Once surface-associated populations are established, reductions remain limited even under cold storage, which has been linked to the protective role of early biofilm formation [39,40]. In addition, the waxy and irregular surface of goldenberry may favor microbial retention and the formation of protected micro-niches that further limit treatment efficacy [41].

Taken together, these results indicate that CFS-based treatments are more effective when applied at early stages, before stable attachment is established, while their impact is reduced once surface-associated populations become more structured and protected. This supports the need for timely postharvest intervention, although further studies incorporating direct biofilm quantification would be required to confirm these mechanisms [31,42,43].

### 3.2. Effect of ExAF-E1 on Goldenberry Microbiota During Storage at Room Temperature

**Figure 3:**
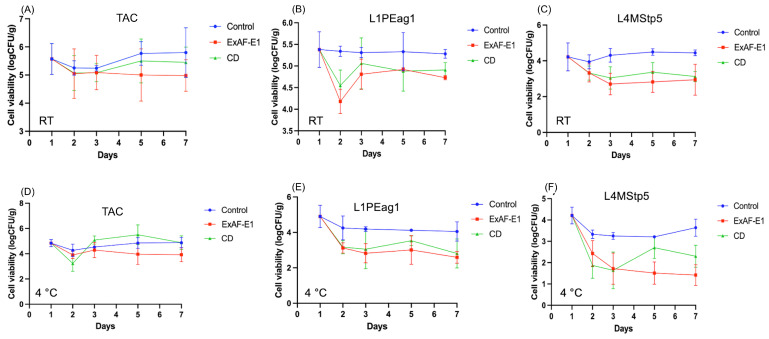
Effect of ExAF-E1 and a commercial disinfectant (CD) on microbial viability of artificially inoculated goldenberry fruits during storage at room temperature (RT) and 4 °C. Panels: (**A**–**C**) show total aerobe counts (TAC) and pathogen-specific populations of *E. coli* L1PEag1 and *S. epidermis* L4MStp5 during storage at RT; (**D**–**F**) present the corresponding data under refrigerated conditions (4 °C). Microbial viability is expressed as log CFU/g over a 7-day storage period. Data are expressed as log CFU/g (mean ± SD, n = 4).

Under RT, TAC in untreated goldenberries remained stable or slightly increased over time, confirming that RT conditions support sustained microbial metabolic activity. In contrast, ExAF-E1 treatment induced an immediate reduction in TAC by day 2 (approximately 0.4–0.5 log CFU/g relative to control), and this suppression was maintained through day 7 with minimal rebound (Figure 3A). The CD treatment showed an initial reduction; however, microbial counts increased at later storage points (days 5–7) and were not significantly different from the control (*p* > 0.05), indicating a reduced persistence of antimicrobial activity compared to ExAF-E1 (*p* < 0.05). These findings indicate that ExAF-E1 exerts rapid antimicrobial activity under conditions favoring active proliferation. Similar antimicrobial responses have been reported in other fruits treated with LAB-derived postbiotics, including strawberries and mango, where reductions in pathogen loads, modulation of surface microbiota, and preservation of fruit quality were observed under storage conditions, supporting the broader applicability of postbiotic-based interventions across fruit matrices [31,44]. Pathogen-specific responses at RT were more pronounced (Figure 3B,C). For L1PEag1, ExAF-E1 produced an early reduction of approximately 1.0 log CFU/g by day 2 compared with the control, followed by partial recovery yet maintaining a consistent 0.5–0.7 log reduction through day 7. CD achieved a more moderate early decline (≈0.5–0.7 log CFU/g), suggesting slower or less sustained inhibition. For L4MStp5, ExAF-E1 demonstrated stronger suppression, achieving reductions approaching 1.2–1.5 log CFU/g during storage, while CD showed comparable or slightly greater reductions at specific time points. Additionally, untreated controls showed stable or marginally growing populations, indicating that antimicrobial intervention rather than environmental constraints was responsible for the reductions seen in treated fruit. The RT data therefore suggest that ExAF-E1 is particularly effective during early storage, when bacterial cells are metabolically active and most susceptible to membrane-disruptive and acidifying metabolites [45]. The maintenance of reduced pathogen levels through day 7 further suggests sustained activity of bioactive compounds within the fruit surface. This pattern indicates a dual mode of action, where the initial log reductions reflect a bactericidal effect, while the sustained suppression of microbial growth without complete elimination suggests a concurrent bacteriostatic activity over storage. The observed reductions are consistent with reports that LAB-derived metabolites, including bacteriocins, organic acids, and low-molecular-weight bioactive compounds, disrupt membrane integrity, promote intracellular acidification, and induce pathogen cell death [46,47,48,49]. In addition, certain LAB metabolites interfere with staphylococcal adhesion and early biofilm formation [49], which may explain the pronounced suppression of L4MStp5 under ambient conditions.

### 3.3. Effect of ExAF-E1 on Goldenberry Microbiota During Cold Storage

Because elevated microbial loads accelerate physicochemical deterioration of fresh produce [8], the ability of ExAF-E1 to suppress pathogen proliferation during refrigerated storage was evaluated. Refrigeration alone reduced TAC across treatments, with control fruit declining by ~0.5–0.8 log CFU/g by day 2 (Figure 3D). ExAF-E1 produced deeper and sustained reductions, reaching ~1.0–1.2 log CFU/g below initial levels by day 7. In comparison, CD induced an initial decrease of ~0.7–1.0 log CFU/g by day 2, followed by partial recovery, with counts increasing by day 5 and approaching or exceeding initial levels by day 7. This pattern indicates that, although CD provides early antimicrobial activity, its effect is less sustained than that of ExAF-E1 under refrigerated conditions.

For L1PEag1, control samples gradually declined by ~0.8–1.0 log CFU/g over 7 days due to cold-induced stress (Figure 3E). ExAF-E1 enhanced this reduction to ~1.5–2.0 log CFU/g relative to baseline and maintained the lowest counts throughout storage. CD treatment resulted in an initial reduction of ~1.5–2.0 log CFU/g by day 2, comparable to ExAF-E1 at early time points; however, this effect was not maintained, with counts increasing by ~0.5–1.0 log CFU/g during subsequent storage, indicating reduced persistence of antimicrobial activity. The strongest treatment effect was observed for L4MStp5 (Figure 3F). Refrigeration alone reduced counts by ~0.8–1.0 log CFU/g. ExAF-E1 induced a continuous decline, reaching ~2.0–2.5 log CFU/g reduction by day 7. CD also produced a marked initial reduction (~1.5–2.0 log CFU/g by day 2), but this was followed by a progressive increase in counts, resulting in higher final populations compared to ExAF-E1.

Overall, while both treatments exhibited antimicrobial activity under refrigeration, ExAF-E1 showed a more stable and sustained inhibitory effect, whereas CD was characterized by strong initial reductions followed by partial regrowth. The progressive reductions observed in ExAF-E1-treated samples indicate a predominantly bactericidal effect under cold conditions, whereas the absence of microbial rebound further supports a bacteriostatic contribution that limits regrowth during storage. However, the sustained decrease in ExAF-E1-treated samples suggests a synergistic interaction between cold stress and metabolite-mediated antimicrobial activity. These findings are consistent with evidence that LAB metabolites interfere with pathogen adhesion and biofilm formation, particularly in *Staphylococcus aureus* [50]. Similar reductions in *E. coli*, Listeria monocytogenes, and *S. aureus* have been reported in strawberries treated with *L. plantarum* extracts under cold storage [51]. Preservation of fruit quality also aligns with previous reports showing that LAB metabolites maintain chemical composition without inducing undesirable fermentation [52]. Comparable antimicrobial effects have been described in mango treated with LAB metabolites [31], supporting the potential of postbiotics as a clean-label preservative for fresh fruit.

The antimicrobial activity of ExAF-E1 likely results from the combined action of LAB-derived metabolites and small bioactive compounds that disrupt cell membranes, induce intracellular acidification, and downregulate virulence pathways [53]. The persistence of inhibition during storage suggests that these extracellular metabolites remain stable on the fruit skin. The waxy and irregular epidermis of goldenberry may facilitate retention of these compounds within surface depressions, promoting localized accumulation and prolonged antimicrobial exposure. In addition to bactericidal effects, several LAB metabolites can disrupt adhesion and early biofilm formation by altering cell surface hydrophobicity or interfering with quorum sensing. This mechanism may contribute to the strong suppression of L4MStp5 observed here, as staphylococcal species depend on surface attachment and biofilm development for persistence on produce surfaces [53]. Together, these mechanisms indicate that ExAF-E1 not only inhibits pathogen growth but also limits the establishment and stability of surface-associated microbial communities.

### 3.4. Multivariate Analysis of Microbiological Dynamics

**Figure 4:**
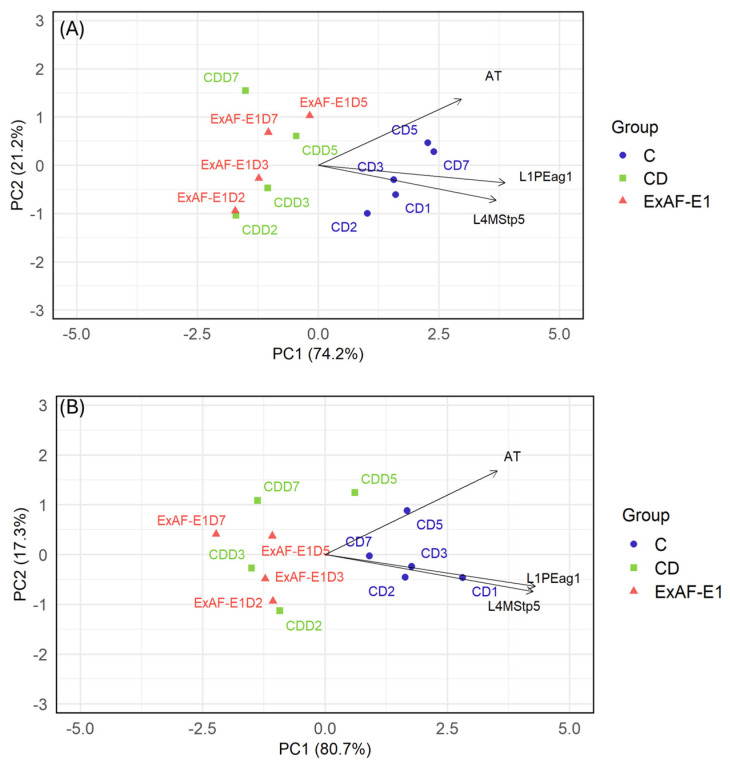
PCA of microbial dynamics in goldenberries under different postharvest treatments. (**A**) RT showing separation of control (C), commercial disinfectant (CD), and ExAF-E1 treatments. (**B**) Storage at 4 °C, illustrating treatment-dependent clustering and the combined influence of refrigeration and antimicrobial treatments. Data points represent sampling times during storage, and vectors indicate the contribution of microbial variables to the principal components.

PCA provided an integrative visualization of treatment effects on microbial dynamics. In this analysis, principal components (PCs) represent linear combinations of the original variables, where PC1 and PC2 capture the largest proportions of total variance. Variable vectors (loadings) indicate the direction and strength of each microbial parameter’s contribution, while the proximity between samples and vectors reflects their association. Under RT (Figure 4A), the first two principal components explained 95.4% of the total variance (PC1 = 74.2%, PC2 = 21.2%). A clear separation among the experimental groups was observed along PC1, with control samples (C) clustering on the positive side and aligning with the vectors of L1PEag1 and L4MStp5, indicating higher contributions of these microbial variables to those samples, while CD samples were distributed on the negative side of PC1. The ExAF-E1- and CD-treated samples progressively shifted toward the center of the factorial plane between days 2 and 7, reflecting reduced microbial burdens (Figure 4A). The early divergence of ExAF-E1-treated samples indicates rapid antimicrobial activity during initial storage, consistent with acidification and bacteriocin-mediated inhibition described for LAB postbiotics [53]. Under refrigeration conditions (Figure 4B), the PCA showed a similar pattern, with PC1 explaining 80.7% and PC2 explaining 17.3% of the variance. The separation between C and CD groups along PC1 remained evident, with control samples again associated with L1PEag1 and L4MStp5. ExAF-E1 samples displayed an intermediate distribution between the two groups. Additionally, PC2 showed diminished contribution of L1PEag1 in ExAF-E1- and CD-treated fruits under refrigeration, supporting synergistic effects between cold stress and metabolite-mediated inhibition. Similar postbiotic efficacy at low temperatures has been described by Rahman et al. [54], who reported reduced bacterial replication and enhanced antimicrobial performance under combined stress conditions. Taken together, temperature clearly modulated antimicrobial dynamics. At RT, treatment effects were most distinguishable during early storage, reflecting rapid inhibition of actively growing cells. Under refrigeration, intrinsic suppression by cold reduced overall microbial growth pressure; however, ExAF-E1 consistently achieved deeper cumulative log reductions, particularly for pathogen-specific populations. Nevertheless, ExAF-E1 maintained antimicrobial activity under cold storage, demonstrating that its bioactive metabolites remain functional across storage environments. The enhanced performance observed at RT suggests that LAB-derived metabolites act more effectively against metabolically active bacterial cells, where membrane-targeting compounds, organic acids, and bacteriocins can exert sustained inhibitory pressure. Under refrigeration, reduced metabolic rates and stress adaptation responses may partially attenuate treatment-dependent variability, although synergistic effects between cold stress and metabolite-mediated inhibition were evident, particularly for L4MStp5. Besides, ExAF-E1 matched or exceeded the efficacy of the commercial disinfectant under several storage conditions while representing a biologically derived alternative. This performance, combined with previously documented preservation of fruit physicochemical quality, supports the application of LAB supernatant as a sustainable postharvest biocontrol strategy.

### 3.5. Influence of Postharvest Treatments on Goldenberry Quality Attributes

#### 3.5.1. Quality Parameters at RT

**Figure 5:**
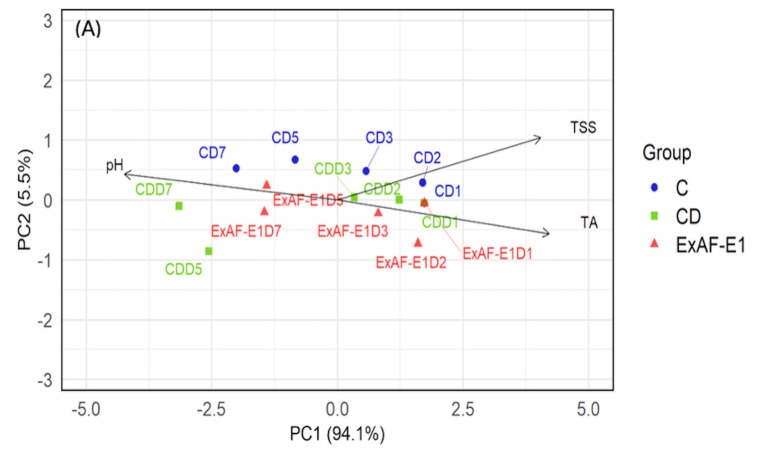

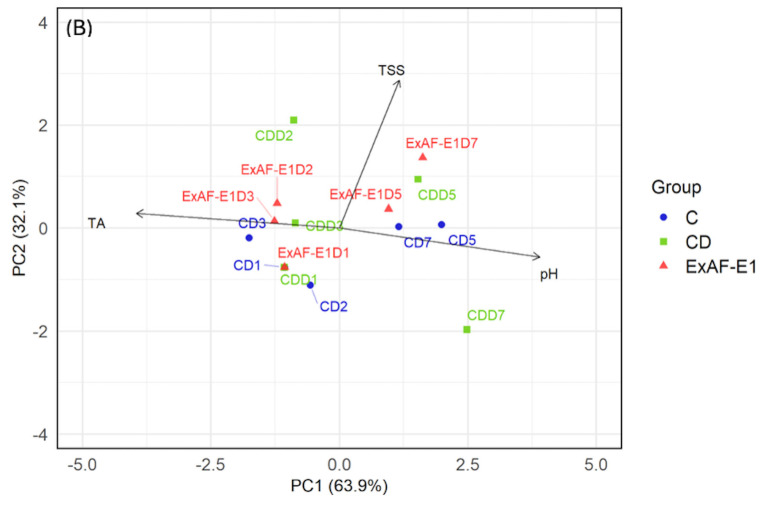
PCA of quality parameters of goldenberry fruits under different treatments. (**A**) PCA based on physicochemical attributes at RT. (**B**) PCA based on physicochemical attributes under 4 °C. TA: titratable acidity; TSS: total soluble solids; CD: commercial disinfectant; ExAF-E1: LAB-derived postbiotic formulation.

During RT storage, goldenberries exhibited physicochemical changes typical of postharvest ripening, characterized by increasing pH and decreasing TA due to organic acid consumption during respiration [55]. In untreated inoculated fruits (C), pH increased from 3.86 ± 0.12 to 4.14 ± 0.11, while TA declined from 1.20 ± 0.15% to 0.97 ± 0.11% by day 7 (Table S1). Similar trends in non-inoculated fruits (WS and UWS) indicate that these changes reflect natural ripening processes rather than microbial activity. Comparable pH increases and acidity declines during goldenberry storage have been reported previously [55]. ExAF-E1-treated fruits maintained the pH and TA values comparable to controls (*p* > 0.05), indicating that the treatment did not alter the natural ripening trajectory. In contrast, CD-treated fruits showed slightly higher pH and lower TA by day 7, suggesting potential interactions between chemical disinfectants and fruit metabolism [52]. TSS remained stable during early storage and declined slightly by day 7, particularly in CD and WS fruits, likely reflecting sugar utilization during respiration or microbial activity. ExAF-E1-treated fruits maintained TSS values comparable to controls, indicating preservation of sweetness. These trends are consistent with reports describing organic acid degradation and carbohydrate metabolism during goldenberry maturation [56]. PCA analysis showed that PC1 explained 94.1% of the variance (Figure 5A), indicating that most variability among samples is captured along this axis, primarily driven by ripening-related parameters (e.g., TSS, TA, pH). Control fruits clustered with higher TSS, reflecting rapid sugar accumulation under ambient storage [1]. ExAF-E1-treated fruits associated with the TA vector, suggesting improved acid preservation, whereas CD samples showed greater dispersion and association with higher pH values [57]. TPC and AOX declined moderately during storage but did not differ significantly among treatments (*p* > 0.05) (Table S2). These changes likely reflect natural phenolic turnover and oxidative processes during senescence [58,59]. Phenolic compounds contribute substantially to antioxidant capacity and plant defense responses [56]. Overall, ExAF-E1 maintained physicochemical and functional attributes while providing antimicrobial control.

#### 3.5.2. Quality Parameters During Cold Storage

The physicochemical properties of goldenberry stored at 4 °C remained stable across treatments, with no significant differences (*p* > 0.05) in pH, TSS, TA, or AAC (Table S3). Values ranged from pH 3.83–4.01, TSS 11.5–11.9 (°Brix), TA 1.10–1.29%, and vitamin C 2.35–3.08 mg/L. Minimal changes were observed in the inoculated control (C) and treated fruits, indicating that refrigeration effectively suppressed metabolic and microbial processes that typically modify acidity and ripening [60]. Stable TSS further suggests limited sugar metabolism and respiration at low temperature [60], while constant TA reflects reduced enzymatic activity and organic acid turnover under cold storage [61]. PCA under refrigeration showed a more balanced distribution of variance (PC1 = 63.9%, PC2 = 32.1%; Figure 5B). pH loaded positively on PC1 and TA negatively, confirming their inverse relationship, while TSS contributed mainly to PC2, indicating limited sugar-driven variability. ExAF-E1 fruits formed a tighter cluster, suggesting improved physicochemical stability when combined with refrigeration. Cold storage is known to suppress respiration and delay ripening, extending shelf life [2], and similar synergistic effects between refrigeration and functional treatments have been reported in other perishable fruits [62]. In contrast, CD samples showed greater dispersion and occasional associations with higher pH or TSS, indicating less controlled preservation. These patterns support the role of ExAF-E1 in maintaining acidity and reducing ripening variability during refrigerated storage [10].

TPC and AOX showed only minor variation during the 7-day period, with no significant differences among treatments (*p* > 0.05) (Table S4). Inoculated controls exhibited a slight decline in TPC and AOX, whereas ExAF-E1 and CD maintained comparable values. Non-inoculated fruits (WS, UWS) initially showed higher TPC but converged with inoculated fruits by day 7, while AOX remained stable. The limited changes suggest that refrigeration slowed oxidative reactions and enzymatic phenolic degradation [58]. Higher initial TPC in unwashed fruits may reflect surface phenolics or stress-induced synthesis at harvest, followed by normal metabolic turnover and phenolic polymerization during storage [63]. Overall, cold storage preserved antioxidant capacity, and ExAF-E1 provided microbial control without compromising phenolic content or fruit quality. From a sensory perspective, the stability of these parameters suggests that major attributes such as color and oxidative-related flavor changes were not adversely affected. However, given that ExAF-E1 contains organic acids and other bioactive metabolites, potential effects on taste and aroma cannot be excluded. Therefore, dedicated organoleptic evaluation is required to confirm consumer acceptance and to determine whether the treatment influences flavor, aroma, or overall sensory perception. These aspects should be addressed in future studies to support the practical application of ExAF-E1 as a postharvest intervention.

#### 3.5.3. Effect of Storage Temperature and Treatments on AAC Stability

**Figure 6:**
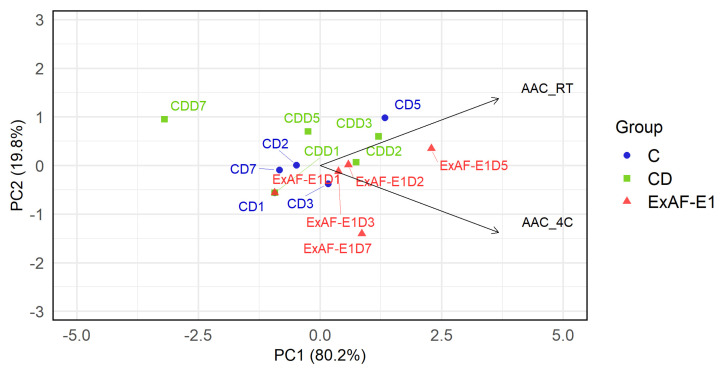
PCA plot of ACC under different treatments and storage conditions. The score plot shows the distribution of samples according to treatments: control (C), commercial disinfectant (CD), and the LAB-derived postbiotic formulation ExAF-E1. The loading vectors represent AAC measured at room temperature (AAC_RT) and under refrigeration (AAC_4C).

AAC did not differ significantly among treatments (*p* > 0.05), although a gradual decline occurred during storage, most evident in CD-treated fruits at day 7 (2.12 ± 0.74 mg/L), whereas ExAF-E1 fruits retained higher levels (3.07 ± 0.27 mg/L) (Table S1), suggesting partial protection against oxidative degradation. Under refrigeration, AAC showed only minor fluctuations without a consistent decline (Table S3), confirming the role of cold storage in limiting enzymatic and oxidative vitamin C losses [60]. PCA revealed that treatment was the primary driver of variability in AAC, with PC1 explaining 80.2% of the total variance (Figure 6). Samples treated with ExAF-E1 clustered along the positive PC1 axis and were closely associated with both AAC_RT and AAC_4C vectors, indicating enhanced preservation of antioxidant capacity compared to control and CD-treated fruits. In contrast, control samples were located on the negative side of PC1, reflecting lower AAC values, while CD samples exhibited a more dispersed and intermediate distribution. PC2 (19.8%) contributed to the separation between AAC_RT and AAC_4C, suggesting a secondary influence of storage temperature on antioxidant responses rather than a dominant effect. This suggests that lower surface microbial loads may limit oxidative degradation by reducing microbial activity and associated reactive oxygen species involved in ascorbic acid depletion [64]. The stabilization observed in ExAF-E1-treated fruits may therefore reflect reduced oxidative stress and microbial pressure, consistent with reports that bioactive postharvest treatments enhance antioxidant preservation [2]. LAB-derived metabolites, including organic acids, exopolysaccharides, and antioxidant peptides, may further contribute by limiting surface oxidation and spoilage [65,66]. In contrast, the broader dispersion of CD samples indicates less consistent protection, possibly due to the lack of antioxidant functionality or stress-related effects on nutrient stability [65]. Further studies across multiple harvest seasons and conditions are needed to confirm the robustness and scalability of these findings.

### 3.6. Ultrastructural Alterations Induced by ExAF-E1

**Figure 7:**
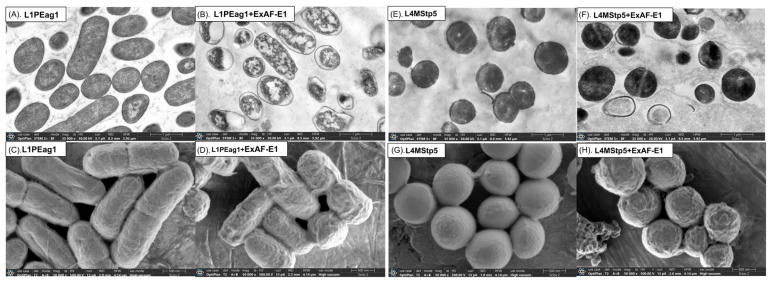
Ultrastructural and surface morphological effects of ExAF-E1 on *E. coli* L1PEag1 and *S. epidermidis* L4MStp5. (**A**,**C**) Untreated *E. coli* L1PEag1 cells showing typical rod-shaped morphology with intact cell envelopes (TEM) and smooth surfaces (SEM). (**B**,**D**) *E. coli* treated with ExAF-E1 exhibiting disrupted cell membranes, irregular morphology, cytoplasmic disorganization (TEM), and surface deformation with wrinkling and collapse (SEM). (**E**,**G**) Untreated *S. epidermidis* L4MStp5 cells displaying characteristic spherical morphology with intact cell walls (TEM) and smooth surfaces (SEM). (**F**,**H**) *S. epidermidis* treated with ExAF-E1 showing altered cell morphology, disrupted intracellular organization (TEM), and rough, damaged surfaces with evidence of structural collapse (SEM). Scale bars: TEM = 1 µm; SEM = 500 nm.

TEM analysis of untreated L1PEag1 cells revealed well-defined rod-shaped morphology, intact cell envelopes, and uniformly electron-dense cytoplasm (Figure 7A), indicating preserved cellular organization. In contrast, ExAF-E1–treated cells exhibited pronounced ultrastructural damage, including disruption of the cell envelope, irregular morphology, and electron-lucent regions indicative of cytoplasmic leakage (Figure 7B). These features are consistent with increased membrane permeability and loss of intracellular contents, which are characteristic of membrane-targeting antibacterial agents [67,68]. SEM results indicate that untreated L1PEag1 cells exhibited smooth and intact surfaces (Figure 7C), whereas treated cells showed significant surface damage, including wrinkling, and deformation (Figure 7D). These morphological changes are indicative of loss of membrane integrity and reduced cellular turgor pressure, commonly associated with bactericidal agents that disrupt lipid bilayers [69].

Untreated *S. epidermidis* L4MStp5 cells displayed typical coccoid morphology with intact peptidoglycan layers and dense cytoplasmic content (Figure 7E). Following ExAF-E1 treatment, cells showed clear structural alterations, including reduced electron density, intracellular disorganization, and morphological distortion (Figure 7F). These observations suggest that ExAF-E1 disrupts both the cytoplasmic membrane and internal cellular organization, even in Gram-positive bacteria. SEM analysis further confirmed the structural damage induced by the ExAF-E1 treatment. Similarly, untreated *S. epidermidis* cells exhibited smooth, spherical morphology (Figure 7G), while ExAF-E1–treated cells displayed roughened surfaces, irregular shapes, and structural deformation (Figure 7H). These findings indicate damage to the cell wall and cytoplasmic membrane, leading to compromised structural stability and potential cell lysis [70].

The combined TEM and SEM observations indicate that ExAF-E1 primarily targets the bacterial cell envelope. In *E. coli*, disruption of the outer membrane likely increases permeability, resulting in leakage of cytoplasmic components and rapid cell death. In *S. epidermidis*, despite the presence of a thick peptidoglycan layer, ExAF-E1 induces significant structural damage, suggesting interaction with the cytoplasmic membrane or destabilization of the cell wall–membrane interface. This mode of action is consistent with broad-spectrum antimicrobial agents that act via membrane permeabilization and structural disruption, rather than specific intracellular targets. Such mechanisms are associated with rapid bactericidal activity and a lower propensity for resistance development [71,72].

### 3.7. Technological Implications for Postharvest Application

From a technological perspective, these results suggest that ExAF-E1 could be implemented as a practical postharvest sanitation treatment for fresh goldenberries. The formulation can be applied through conventional washing or spray systems already used in fresh-produce packing lines, facilitating integration without major infrastructure modifications. The antimicrobial activity observed under both RT and refrigerated storage further indicates compatibility with cold-chain distribution systems commonly used for export fruits. Because ExAF-E1 consists of cell-free microbial metabolites rather than viable microorganisms, it may provide advantages in stability, safety, and regulatory acceptance compared with live microbial biocontrol agents [73]. Postbiotic preparations are increasingly recognized as natural antimicrobial agents in food systems and have shown potential to inhibit spoilage and pathogenic microorganisms through metabolites such as organic acids, bacteriocins, and antimicrobial peptides [74]. Their application in food preservation aligns with current industry efforts to develop clean-label and sustainable alternatives to synthetic chemical sanitizers [75]. Consequently, LAB-derived postbiotic formulations such as ExAF-E1 represent a promising strategy for enhancing microbial safety while maintaining the quality of highly perishable fruits during postharvest storage.

However, several limitations should be acknowledged. Although the CFS composition was characterized and includes multiple bioactive compounds, such as peptides with predicted antimicrobial activity [23,53], their individual and synergistic contributions to the overall effect remain unresolved. In addition, the translation of these findings into a standardized, scalable formulation requires further optimization to ensure consistency, stability, and efficacy under industrial conditions. Future work should therefore focus on validation in real postharvest systems, as well as on regulatory assessment and evaluation of commercial viability, including feasibility for large-scale production and market adoption.

## 4. Conclusions

This study highlights the potential of LAB-derived postbiotics as a viable, clean-label strategy for improving the microbial safety of fresh produce. The consistent performance of ExAF-E1 across storage conditions supports its applicability within postharvest systems, particularly where stable and non-viable antimicrobial solutions are preferred. These findings reinforce the importance of early intervention in contamination control and demonstrate how biologically derived compounds can complement or replace conventional chemical disinfectants. However, this work is limited by its laboratory-scale design and the absence of direct mechanistic analyses (e.g., biofilm quantification or molecular responses), which constrains definitive interpretation of microbial adaptation processes. Future research should focus on validation under commercial supply chain conditions, integration into existing processing lines, and evaluation of sensory quality and consumer acceptance. In addition, mechanistic studies (e.g., transcriptomic or proteomic approaches), broader pathogen coverage, and predictive shelf-life modeling would further strengthen the applicability and scalability of this approach for fresh fruit preservation and safety management.

## Data Availability

The original contributions presented in this study are included in the article/Supplementary Materials. Further inquiries can be directed to the corresponding author.

## Supplementary Materials

The following supporting information can be downloaded at https://www.mdpi.com/article/10.3390/foods15101830/s1. Table S1. Physicochemical quality parameters of goldenberry during storage at room temperature under different treatments; Table S2. Total phenolic content (TPC) and antioxidant capacity (AOX) of goldenberry under different treatments during storage at room temperature; Table S3. Physicochemical parameters and vitamin C content of goldenberry fruits stored at 4 °C under different postharvest treatments under cold conditions; Table S4. Total phenolic content (TPC) and antioxidant capacity (AOX) of goldenberry under different treatments during storage under cold conditions.
