# Supplementary material for: Dual Lactiplantibacillus plantarum-Derived Postbiotics Reduce Pathogens and Preserve the Quality of Goldenberry (Physalis peruviana L.) During Storage
Source: Foods. 2026 May 21;15(10):1830. doi: 10.3390/foods15101830 (PMC13206692; doi:10.3390/foods15101830)
Supplement: Supplementary file 1 [file foods-15-01830-s001.zip › foods-4297146-supplementary.pdf]

Supplementary materials

Dual *Lactiplantibacillus plantarum*-Derived Postbiotics Reduce Pathogens and Preserve the Quality of Goldenberry (*Physalis peruviana* L.) During Storage

Diana Molina, Pamela Reyes, Yuleissy Cuamacas , Evelyn Angamarca, Clara Ortega, Renato Centeno and Gabriela N. Tenea *

Biofood and Nutraceutics Research and Development Group, Faculty of Engineering in Agricultural and Environmental Sciences, Universidad Tecnica del Norte, Ibarra 100105, Ecuador; dmolinap@utn.edu.ec (D.M.); pcreyes@utn.edu.ec (P.R.); yncuamacasc@utn.edu.ec (Y.C.); elangamarca@utn.edu.ec (E.A.);
cgortegab@utn.edu.ec (C.O.); rxcentenoa@utn.edu.ec (R.C.)
* Correspondence: gntenea@utn.edu.ec

Table S1. Physicochemical quality parameters of goldenberry during storage at room temperature under different treatments.

Code	Storage time (day)	pH	Total soluble solids (TSS)	Titratable acidity (%)	Vitamin C (mg ascorbic acid/L)
C	1	3,86±0,12^{abc}	11,63±0,78^{a}	1,2±0,15^{a}	2,68±0,64^{a}
C	2	3,88±0,09^{abcd}	11,72±0,90^{a}	1,2±0,14^{abc}	2,66±0,63^{a}
C	3	3,96±0,13^{abcde}	11,57±0,80^{a}	1,11±0,14^{abc}	2,81±0,34^{a}
C	5	4,05±0,13^{abcde}	11,36±0,83^{a}	1,01±0,14^{abc}	2,78±0,35^{a}
C	7	4,14±0,11^{de}	11,12±0,39^{a}	0,97±0,11^{a}	2,62±0,69^{a}
ExAF-E1	2	3,85±0,10^{ab}	11,43±0,60^{a}	1,23±0,21^{abc}	2,81±0,46^{a}
ExAF-E1	3	3,91±0,11^{abcd}	11,42±0,82^{a}	1,16±0,14^{abc}	2,81±0,29^{a}
ExAF-E1	5	4,06±0,15^{bcde}	11,15±0,71^{a}	1±0,10^{abc}	3,02±0,25^{a}
ExAF-E1	7	4,07±0,09^{bcde}	11,02±0,63^{a}	1,03±0,09^{abc}	3,07±0,27^{a}
CD	2	3,91±0,11^{abcd}	11,56±0,72^{a}	1,19±0,16^{abc}	2,83±0,62^{a}
CD	3	3,96±0,12^{abcde}	11,41±0,65^{a}	1,12±0,11^{abc}	2,82±0,44^{a}
CD	5	4,1±0,13^{cde}	10,65±0,52^{a}	0,98±0,09^{ab}	2,59±0,40^{a}
CD	7	4,2±0,15^{e}	10,75±0,83^{a}	0,94±0,11^{a}	2,12±0,74^{a}
UWS	0	3,84±0,14ab	11,64±0,71^{a}	1,2±0,23^{abc}	2,52±0,80^{a}
UWS	7	4,06±0,14^{bcde}	10,91±0,79^{a}	1,01±0,09^{abc}	2,42±0,42^{a}
WS	0	3,8±0,14^{a}	11,42±0,49^{a}	1,29±0,19^{bc}	2,45±0,64^{a}
WS	1	3,81±0,13^{a}	11,41±0,49^{a}	1,3±0,25^{c}	2,72±0,61^{a}
WS	7	4,06±0,15^{bcde}	10,62±0,66^{a}	1,01±0,10^{abc}	2,3±0,54^{a}

Values represent mean ± standard deviation (SD) of pH, total soluble solids (TSS), titratable acidity (%), and vitamin C (mg ascorbic acid/L) measured at different storage times (days). Treatments: C (control), ExAF-E1 (LAB-derived postbiotics formulation), CD (commercial disinfectant), UWS (unwashed samples), and WS (washed samples). Different lowercase letters within the same column indicate significant differences among treatments and storage times ($p < 0.05$) according to Tukey's multiple comparison test.

Table S2. Total phenolic content (TPC) and antioxidant capacity (AOX) of goldenberry under different treatments during storage at room temperature.

Code	Storage time (day)	TPC (mgGAE/ml)	AOX(umol Trolox/ml)
C	1	2,77±0,43ª	228,57±15,18ª
C	7	2,58±0,38ª	218,75±11,87ª
ExAF-E1	1	2,77±0,43ª	228,57±15,18ª
ExAF-E1	7	2,62±0,53ª	223,96±14,81ª
CD	1	2,77±0,43ª	228,57±15,18ª
CD	7	2,81±0,52ª	224,98±17,05ª
UWS	0	3,38±0,81ª	221,47±19,86ª
UWS	7	2,84±0,58ª	225,98±18,25ª
WS	0	3,7±0,94ª	220,99±19,64ª
WS	1	3,38±0,94ª	221,21±14,47ª
WS	7	2,77±0,55ª	225,38±18,54ª

Values are expressed as mean ± standard deviation (SD) of total phenolic content (mg GAE/mL) and antioxidant capacity (μmol Trolox/mL) at the indicated storage times (days). Treatments: C (control), ExAF-E1 (LAB-derived postbiotics formulation), CD (commercial disinfectant), UWS (unwashed samples), and WS (washed samples). Different lowercase letters within the same column indicate significant differences among treatments and storage times ($p < 0.05$) according to Tukey's multiple comparison test. Identical letters denote no statistically significant differences.

Table S3. Physicochemical parameters and vitamin C content of goldenberry fruits stored at 4 °C under different postharvest treatments under cold conditions.

Code	Storage time (day)	pH	Total soluble solids (TSS)	Titratable acidity (%)	Vitamin C (mg ascorbic acid/L)
C	1	3,86±0,10ª	11,53±0,44ª	1,25±0,15ª	2,46±0,75ª
C	2	3,88±0,13ª	11,51±0,22ª	1,23±0,14ª	2,61±0,37ª
C	3	3,83±0,12ª	11,58±0,49ª	1,27±0,19ª	2,65±0,38ª
C	5	3,92±0,15ª	11,7±0,51ª	1,10±,13ª	3,03±0,28ª
C	7	3,92±0,13ª	11,68±0,45ª	1,17±0,12ª	2,54±0,33ª
ExAF-E1	2	3,85±0,14ª	11,67±0,35ª	1,27±0,08ª	2,77±0,45ª
ExAF-E1	3	3,84±0,14ª	11,63±0,70ª	1,25±0,13ª	2,72±0,34ª
ExAF-E1	5	3,91±0,11ª	11,71±0,84ª	1,17±0,17ª	3,08±0,19ª

ExAF-E1	7	3,94±0,07^{a}	11,85±0,44^{a}	1,17±0,12^{a}	2,6±0,40^{a}
CD	2	3,85±0,13^{a}	11,87±0,41^{a}	1,27±0,11^{a}	2,8±0,55^{a}
CD	3	3,85±0,12^{a}	11,63±0,45^{a}	1,24±0,20^{a}	2,95±0,23^{a}
CD	5	3,93±0,11^{a}	11,79±0,49^{a}	1,16±0,15^{a}	2,75±0,22^{a}
CD	7	4,01±0,11^{a}	11,48±0,55^{a}	1,13±0,16^{a}	2,35±0,57^{a}
UWS	0	3,84±0,14^{a}	11,64±0,71^{a}	1,2±0,23^{a}	2,52±0,80^{a}
UWS	7	3,91±0,14^{a}	11,46±0,49^{a}	1,16±0,19^{a}	2,36±0,76^{a}
WS	0	3,8±,14^{a}	11,42±0,71^{a}	1,29±0,23^{a}	2,45±0,80^{a}
WS	1	3,81±0,11^{a}	11,51±0,44^{a}	1,27±0,21^{a}	2,46±0,68^{a}
WS	7	3,93±0,19^{a}	11,49±0,45^{a}	1,21±0,22^{a}	2,4±0,73^{a}

Values represent mean ± standard deviation (SD) of pH, total soluble solids (TSS), titratable acidity (%), and vitamin C (mg ascorbic acid/L) measured at different storage times (days). Treatments: C (control), ExAF-E1 (LAB-derived postbiotics formulation), CD (commercial disinfectant), UWS (unwashed samples), and WS (washed samples). Different lowercase letters within the same column indicate significant differences among treatments and storage times ($p < 0.05$) according to Tukey's multiple comparison test.

Table S4. Total phenolic content (TPC) and antioxidant capacity (AOX) of goldenberry under different treatments during storage under cold conditions.

**Code**	**Storage time (day)**	**TPC (mgGAE/ml)**	**AOX(umol Trolox/ml)**
C	1	2,94±1,00a	230,97±10,99^{a}
C	7	2,62±0,53^{a}	223,96±14,81^{a}
ExAF-E1	1	2,94±1,00a	230,97±10,99^{a}
ExAF-E1	7	2,81±0,53^{a}	224,98±17,05^{a}
CD	1	2,94±1,00a	230,97±10,99^{a}
CD	7	2,91±0,52^{a}	227,22±16,86^{a}
UWS	0	3,38±0,81^{a}	221,47±19,86^{a}
UWS	7	2,44±0,55^{a}	230,88±18,54^{a}
WS	0	3,7±0,81^{a}	220,99±19,86^{a}
WS	1	3,39±0,96^{a}	223,54±11,41^{a}
WS	7	2,75±1,23^{a}	224,6±21,90^{a}

Values are expressed as mean ± standard deviation (SD) of total phenolic content (mg GAE/mL) and antioxidant capacity (μmol Trolox/mL) at the indicated storage times (days). Treatments: C (control), ExAF-E1 (LAB-derived postbiotics formulation), CD (commercial disinfectant), UWS (unwashed samples), and WS (washed samples). Different lowercase letters within the same column indicate significant differences among treatments and storage times ($p < 0.05$) according to Tukey's multiple comparison test. Identical letters denote no statistically significant differences.
